# Perinatal specimens of *Maiasaura* from the Upper Cretaceous of Montana (USA): insights into the early ontogeny of saurolophine hadrosaurid dinosaurs

**DOI:** 10.7717/peerj.4734

**Published:** 2018-05-17

**Authors:** Albert Prieto-Marquez, Merrilee F. Guenther

**Affiliations:** 1 Mesozoic Research, Catalan Institute of Paleontology Miquel Crusafont, Sabadell, Spain; 2 Elmhurst College, Elmhurst, IL, United States of America

**Keywords:** Evolution, Dinosaur, Cretaceous, Growth, Ontogeny, Anatomy, Hadrosaurid

## Abstract

Perinatal specimens of hadrosaurids discovered in the late 1970’s by field crews from Princeton University were significant in providing evidence of the early ontogenetic stages in North American dinosaurs. These specimens from the Campanian (Upper Cretaceous) Two Medicine Formation of Montana consist of over a dozen skeletons referable to the saurolophine hadrosaurid *Maiasaura peeblesorum*, but never fully figured or described. Here, we provide a more complete documentation of the morphology of these specimens, along with an examination of variation during a large span of the development of saurolophine hadrosaurids. Many ontogenetic changes in the available facial and mandibular elements are associated with the progressive elongation of the preorbital region of the skull and mandible. In the postcranium, limb bones change nearly isometrically, with exception of certain elements of the forelimb. Some cranial and postcranial characters commonly used for inferring hadrosaurid phylogenetic relationships remain invariable during the ontogeny of *M. peeblesorum*. This indicates that early ontogenetic stages may still provide a limited amount of character information useful for systematics and phylogenetic inference.

## Introduction

Analysis of how dinosaur skeletal morphology changed during growth is instrumental for understanding the paleobiology and evolution of dinosaurs. Studies on heterochrony and life history benefit from an identification of ontogenetic stages, which in turn rely on proper use of ontogenetically dependent characters.

Specimens representing the earliest ontogenetic stages in dinosaurs have been recovered for a number of taxa (Horner & Currie, 1994; Norell et al., 1994; Mateus et al., 1997; Chiappe et al., 1998; Horner, 1999; Reisz et al., 2005; Grigorescu & Csiki, 2006; Sullivan, Lucas & Jasinski, 2011; Wosik, Goodwin & Evans, 2018). However, the discovery of the hadrosaurid *Maiasaura peeblesorum*, particularly the finding of perinatal specimens referred to this species, was instrumental in providing evidence of dinosaur reproductive behavior and parental care (Horner, 1982; Horner, 1983; Horner, 1999; Horner & Makela, 1979). Equally significant, the collection of *M. peeblesorum* specimens has contributed substantially to a large data set rich in both numbers of individuals and diverse representation of growth stages. Because *M. peeblesorum* includes one of the best-represented ontogenetic series available for the Hadrosauridae, the combined data from adults and earlier growth stages provided numerous opportunities to further knowledge on hadrosaurid paleohistology (Horner, De Ricqlès & Padian, 1999; Horner, De Ricqlès & Padian, 2000; Horner, De Ricqlès & Padian, 2001), life history and population biology (Kilbourne & Makovicky, 2010; Erickson, Curry Rogers & Yerby, 2001; Woodward et al., 2015), biomechanics (Dilkes, 2000; Dilkes, 2001), and comparative ontogenies (Guenther, 2009; Guenther, 2014). These studies led to the development of research techniques and paleobiological insights with broad applications throughout the Dinosauria.

*Maiasaura peeblesorum* is a saurolophine (Prieto-Márquez, 2010) hadrosaurid and a member of the tribe Brachylophosaurini (Gates et al., 2011). This species was named and described by Horner & Makela (1979) on the basis of a partial skull and mandible collected from Campanian strata of the Two Medicine Formation cropping out near the town of Choteau, Teton County, northwestern Montana. *M. peeblesorum* is characterized by a wide elongate rostrum, a long thin and steeply angled quadradojugal process of the jugal, and a short solid nasofrontal crest that projects rostrodorsally medial to the orbit (Horner & Makela, 1979; Horner, 1983). While numerous specimens of this species have been collected over the past decades and have been used in multiple studies, the osteology of the original perinatal individuals has not been fully documented.

We aim to provide a more detailed understanding of the morphological changes that took place in the earlier growth stages of the cranial and postcranial skeleton of *Maiasaura peeblesorum* in particular and saurolophines in general. This is accomplished through detailed documentation of the anatomy of the assemblage of perinatal specimens of *M. peeblesorum* reported in the original paper by Horner & Makela (1979), emphasazing those morphological attributes that are variable through ontogeny.

## Systematic Paleontology

**Table:** 

Dinosauria Owen, 1842
Ornithischia Seeley, 1887
Ornithopoda Marsh, 1881
Iguanodontia Dollo, 1888
Hadrosauridae Cope, 1870
Saurolophinae Brown, 1914, sensu Prieto-Márquez, 2010
Brachylophosaurini Gates et al., 2011
*Maiasaura peeblesorum* Horner & Makela, 1979

### Referred material

The perinatal specimens are housed at the YPM under a single catalogue number, YPM-PU 22400, and include: six left and eight right maxillae (as well as three maxillary fragments), two partial left quadrates, two right jugals, 12 left and 12 right dentaries (as well as two dentary fragments), a partial left sternal plate, a right coracoid, partial left and right scapulae, six left and six right humeri, two right and two left ulnae, two right and one left radii, left manus, right manual digits II, III and IV, a nearly complete right ilium missing the postacetabular process, the central plate of a right ilium, the preacetabular processes of a left and a right ilia, one right and two left proximal ischiadic fragments, a left pubis, 24 femora, 16 tibiae, a partial left and three right fibulae, a left and right astragalus, a left calcaneum, a right metatarsal II, two left and one right metatarsal III, a right metatarsal IV, an articulated left and right pes, a pedal phalanx III-1, and various other indeterminate postcranial fragments.

### Locality and horizon

The specimens were collected in 1978 by JR Horner, R Makela and field crew members from Princeton University in 1978 from strata of the Campanian Two Medicine Formation, Willow Creek Anticline, Teton County, Montana.

### Remarks

In their original paper, Horner & Makela (1979: p. 298) stated that the perinatal skulls agree with that of adult *Maiasaura peeblesorum* in having “short, wide deflected premaxillary bills, long shallow maxillae with anterior maxillary processes and notches, and long, thin and steeply angling quadratojugal processes of the jugals”. We were unable to locate a premaxilla among the perinate remains. However, as the preorbital region of the skull lengthens during ontogeny in hadrosaurids (Prieto-Márquez, 2011; Prieto-Márquez, 2014) it is unlikely that the perinate premaxillary bill would be of the same length as that of adults. In relation to this lengthening of the preorbital region of the skull, the maxillae of the perinates is craniocaudally shortened in comparison to that of adults and therefore, appears substantially different (see maxilla description below). The rostral maxillary process and notch of the maxilla mentioned by Horner & Makela (1979) probably refers to the rostromedial process and the notch existing between this process and the contiguous rostroventral apex of this bone. As described by Horner & Makela (1979), these are attributes present in all saurolophine hadrosaurids and do not characterize *M. peeblesorum*. The jugal, however, offers two characters that in combination could be used to support referral of the perinates to *M. peeblesorum*. One of these characters was included by Horner & Makela (1979) in the diagnosis of *M. peeblesorum* and consists of a steeply angled quadratojugal flange. An additional condition is the prominent caudoventral flange that reaches a depth that is more than 1.55 times the depth of the caudal constriction, that, although it is not exclusive of *M. peeblesorum*, it is present in this species as well as in other brachylophosaurines (Prieto-Márquez, 2010). Notably, as described below, perinates display a plantar ridge in pedal unguals. Such a ridge has only been observed in *M. peeblesorum* and the closely related *Brachylophosaurus canadensis* (Prieto-Márquez, 2005). Taken in combination, these characters of the jugal and pedal unguals, along with the close proximity (“found within 100 m of the nest” according to Horner & Makela, 1979) of the perinatal remains and those of the type specimen of *M. peeblesorum* (YPM-PU 22405), lead us to maintain the original referral of YPM-PU 22400 to this species.

**Figure 1:**
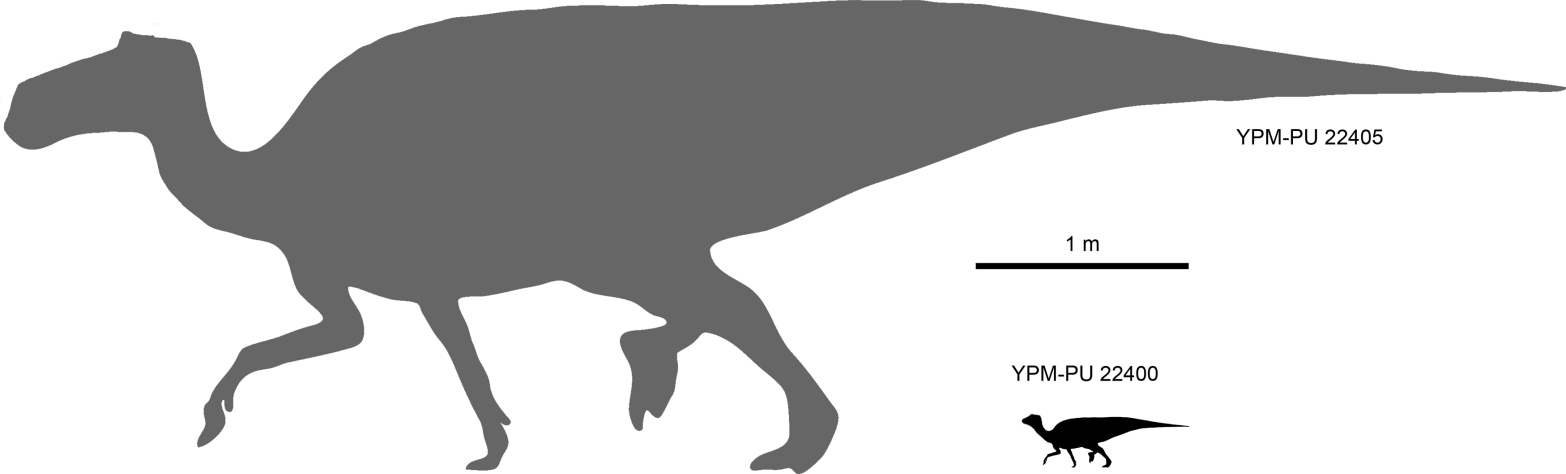
Size comparison between the perinatal individuals in YPM-PU 22400 and the presumably adult type specimen YPM-PU 44505. Adult length estimation from Horner, De Ricqlès & Padian (2000). Perinate scaled down from the adult based on femoral length comparison.

For the purpose of this study and in order to establish a size reference, the largest known specimens (OTM F138, ROM 44770, TCMI 2001.89.2, YPM-PU 22405) of *Maiasaura peeblesorum* are regarded as indicative of adult size (Fig. 1).

**Table:** 

PERINATAL OSTEOLOGY OF *MAIASAURA PEEBLESORUM*

### Cranial elements

**Figure 2:**
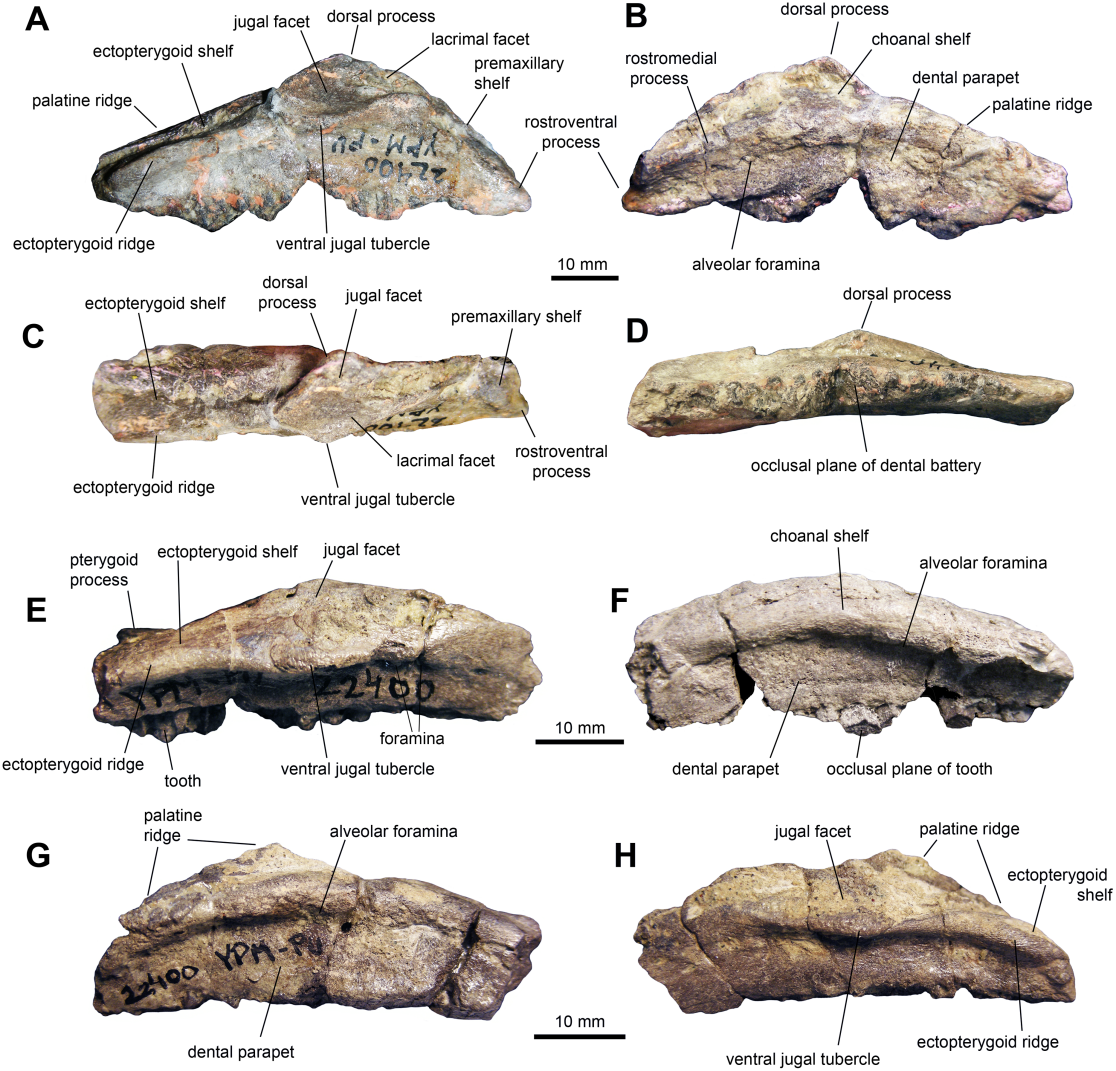
Maxillae of perinatal *Maiasaura peeblesorum* (YPM-PU 22400). (A–D) Right maxilla missing parts of the dentral battery in lateral, medial, dorsal, and ventral views, respectively. (E) and (F) Right maxilla missing the rostral end in lateral and medial views, respectively. (G) and (H) Left maxilla missing the rostral end in medial and lateral views, respectively. Photographs by Albert Prieto-Marquez.

*Maxilla:* The maxilla shows triangular lateral and medial profiles, caused by steep premaxillary and ectopterygoid shelves (Figs. 2A and 2B; Table 1). The rostroventral process and adjacent premaxillary shelf form a 40-degree angle with the tooth row. The lateral surface of the rostrodorsal region of the maxilla is triangular and rostrocaudally abbreviated with relatively little elevation. On the medial side, there is a thick and prominent rostromedial process that converges ventrally to meet the rostroventral process of the maxilla. Both processes enclose a triangular groove that opens caudally to end medial to the dorsal process. The rostral segment of the rostromedial process is missing in all maxillae. The jugal articular surface extends longitudinally throughout 30 percent of the length of the maxilla and accounts for about half of the depth of the lateral surface of the bone. This facet is bounded ventrally by a thick ridge leading caudoventrally to a prominent ventral jugal tubercle. Two maxillary foramina are present below that ridge and additional foramina may be present further rostrally, variably observed among the specimens. When preserved, the dorsal process is missing its dorsal border, and shows an elongated lacrimal facet. Caudal to the jugal joint, a relatively large ectopterygoid shelf extends caudoventrally. This region accounts for 40 percent of the length of the maxilla and represents the widest portion of bone, contrasting with the mediolaterally compressed rostral region. This shelf is bordered medially by a palatine ridge that extends mediodorsally (Fig. 2H) and laterally by a thick ectopterygoid ridge (Fig. 2E). Both the ectopterygoid shelf and the palatine ridge are inclined to form an angle of 20 degrees with the tooth row. A stubby pterygoid process projects caudally from the caudomedial end of the maxilla. On the medial surface of the element, a gently arcuate row of alveolar foramina lies immediately dorsal to the dental parapet, about the level of the dorsal third of the depth of the maxilla at mid-length (Fig. 2G).

**Table 1:** Selected cranial measurements (in mm) of the YPM-PU 22400 perinatal specimens of *Maiasaura peeblesorum*. Measurements are taken on the most complete maxilla (Figs. 2A–2D), jugal (Fig. 3D), quadrate (Fig. 3C), and dentary (Figs. 4A–4C). All other maxillae, jugal, quadrate, and dentaries are of a size similar to that of the measured bones.

**Element**	**Measurement**
Maxilla, total length	60
Maxilla, height from ventral margin to apex of dorsal process (incomplete)	20
Maxilla, length of ectopterygoid shelf	25
Maxilla, maximum width across ectopyerygoid shelf	14
Jugal, length from incomplete rostral process to incomplete quadratojugal flange	42
Jugal, height from ventral margin to incomplete apex of postorbital process	23
Quadrate, height from lateral condyle to articular head	61
Dentary, total length	58
Dentary, dental battery length	49
Dentary, maximum dental battery depth	22
Dentary, maximum depth of mandibular ramus	19
Dentary, width at mid-length of the dental battery	12

**Figure 3:**
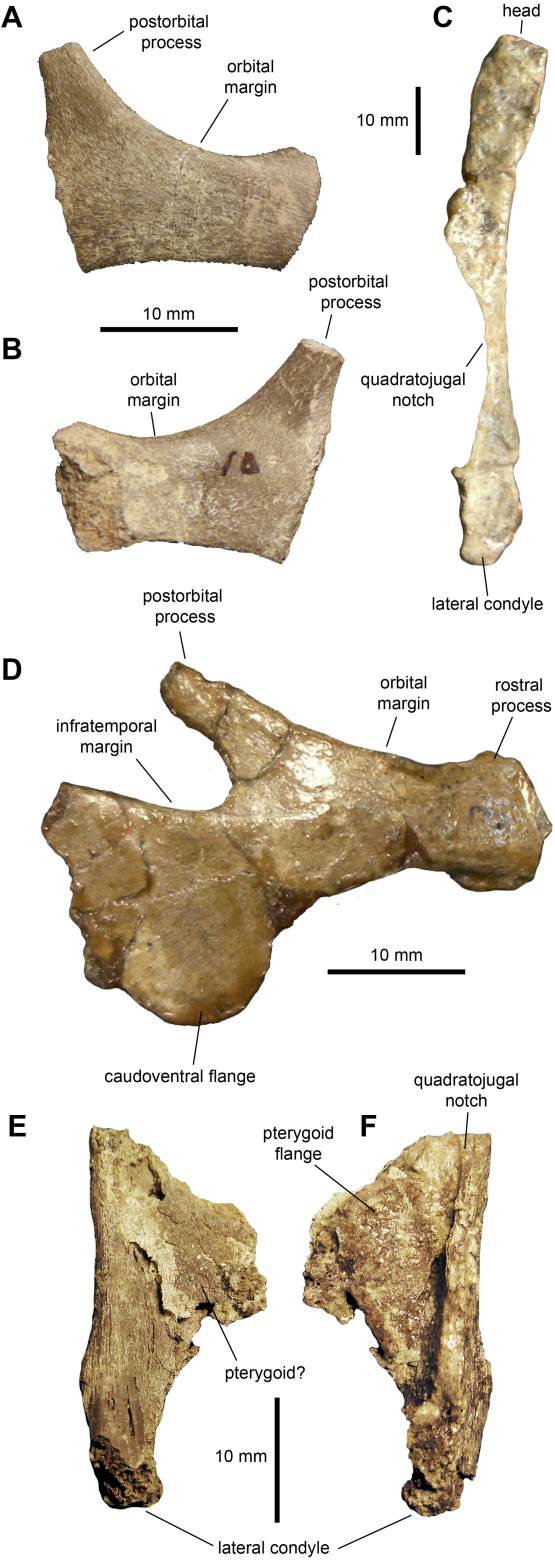
Facial elements of *Maiasaura peeblesorum* perinates (YPM-PU 22400). (A & B) Partial right jugal in lateral and medial views, respectively. (C) Partial left quadrate in lateral view. (D) Left jugal in lateral view. (E & F) Ventral half of right quadrate in lateral and medial views, respectively. Photographs by Albert Prieto-Marquez.

*Jugal:* The jugal (Figs. 3A, 3B and 3D; Table 1) displays an extensive orbital margin that is substantially wider than the infratemporal margin. The great breadth of the orbital margin is caused by the strong caudal inclination of the postorbital process, that forms a 140-degree angle with the long axis of the rostral process. The rostral process is missing the apex in the available jugals. The ventral margin of the rostral process is broad and rounded. Beneath the infratemporal margin, the caudal constriction of the jugal is 1.3 times the depth of the rostral constriction. The caudoventral flange is D-shaped and very broad. The maximum depth from the infratemporal margin to the caudoventral margin of the flange is 1.57 times the depth of the caudal constriction.

*Quadrate:* The quadrate is a long and slender element (Fig. 3C and Table 1). The bone is nearly straight, with a gentle caudal tilt along the caudal margin of its dorsal third. The quadratojugal notch forms a long and wide embayment on the cranial margin of the quadrate. As preserved, however, the breadth of the quadratojugal notch appears magnified by breakage of its dorsal and, particularly, ventral margins in the more complete quadrate (Fig. 3C). The mid-length of the quadratojugal notch is positioned slightly ventral to the mid-length of the quadrate. On the medial side, a wide pterygoid flange extends rostromedially (Figs. 3E and 3F). An irregular fragmentary lamina that appears attached to the medial surface of the flange may represent a remnant of the quadrate wing of the pterygoid (Fig. 3E). The ventral condyles display the typical hadrosaurid triangular ventral profile.

**Figure 4:**
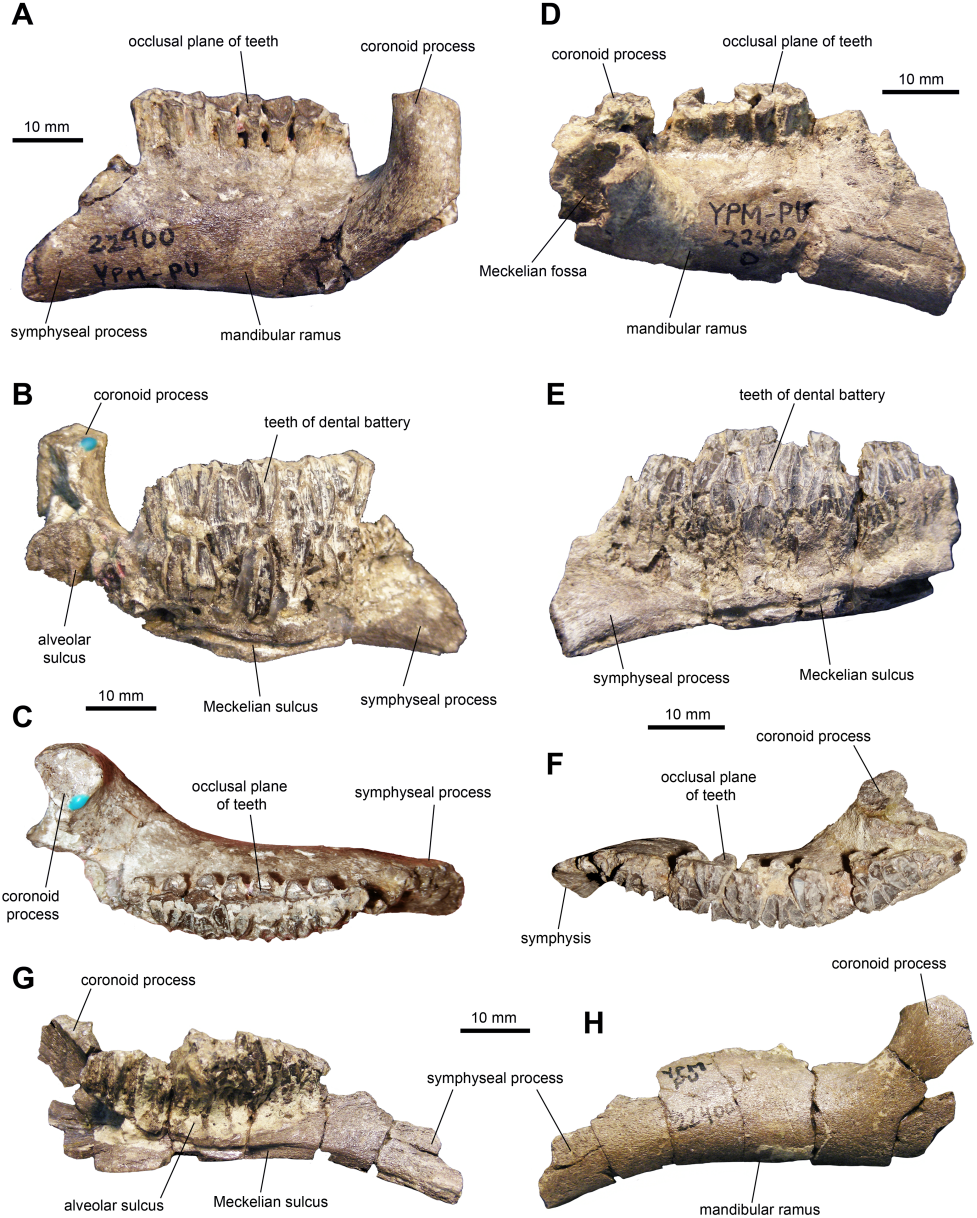
Mandibular elements of *Maiasaura peeblesorum* perinatal specimens (YPM-PU 22400). (A–C) Left dentary in lateral, medial, and dorsal views. (D–F) Right dentary in lateral, medial, and dorsal views. (G) and (H) Left dentary in medial and lateral views, respectively. Photographs by Albert Prieto-Marquez.

*Dentary:* This is one of the most common elements in the sample of specimens (Fig. 4 and Table 1). The mandibular ramus is short and deep. The ventral margin of the dental battery is deeply arcuate in lingual view. The coronoid process is remarkably thick and well offset laterally, leaving a broad shelf between its base and the tooth battery. The dorsal end of the coronoid process is further expanded rostrally and its medial surface bears numerous fine striations. These striations are oriented rostroventrally relative to the vertical axis of the process. The dental battery ends caudal to the coronoid process, as in all adult hadrosaurids (Prieto-Márquez, 2010). A broad channel extends from the coronoid process ventrally and rostrally to form a sulcus that underlies the dental battery. In occlusal view, the tooth row is laterally bowed in some specimens (Fig. 4F) but appears straight in other individuals (Fig. 4C). Rostrally, the dentary ends in a short symphyseal process, the ventral margin of which forms an angle with the long axis of the tooth row that varies between 12 and 20 degrees among the specimens.

**Figure 5:**
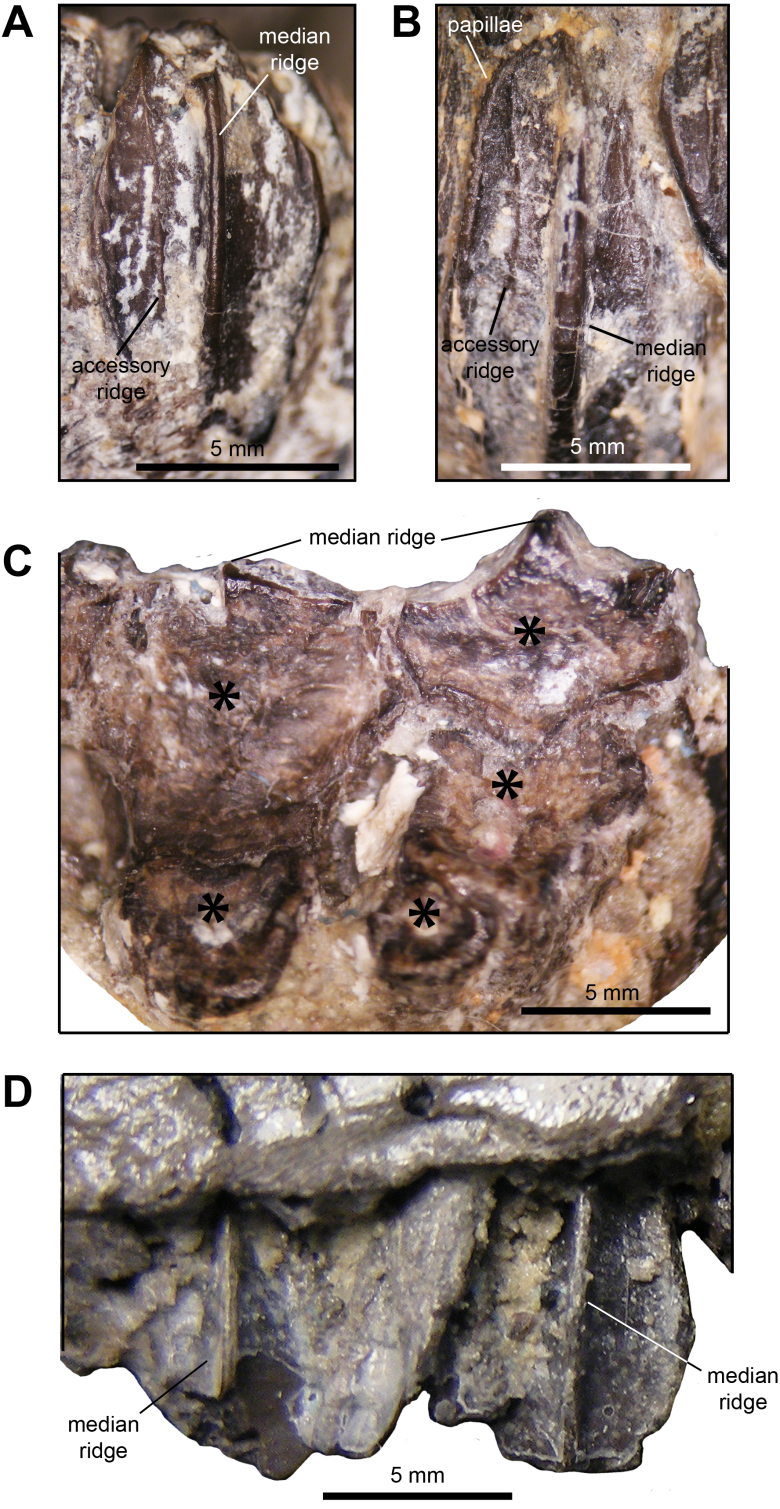
Dentition of *Maiasaura peeblesorum* perinatal specimens (YPM-PU 22400). (A) and (B) Dentary tooth crowns in mesial view. (C) Occlusal surface of dentary teeth. (D) Maxillary tooth crowns in labial view. Photographs by Albert Prieto-Marquez.

*Dentition:* The dentaries contain between ten and 12 tooth positions and there are a maximum of three teeth per alveolus at the deepest point of the dental battery. Generally, the dentary occlusal plane consists of two tooth crowns arranged labiolingually at mid-length of the dental battery; however, in a few cases a small portion of a third crown is present at the lingual border of the occlusal plane (Fig. 5C). As observed in brachylophosaurins (e.g., *Brachylophosaurus canadensis* FMNH 862, *Maiasaura peeblesorum* OTM F138), these teeth form a labiolingually concave occlusal plane, produced by the occlusal surface of the teeth meeting at an angle of 120 degrees. Dentary tooth crowns are relatively broad, with a height/width ratio of 2.3. The labial surface of these crowns bear a prominent median ridge, variably flanked by one or two finer and slightly sinuous accessory ridges (Figs. 5A and 5B). Marginal papillae are diminutive, knobby, and widely spaced structures.

The maxillae display a maximum 15 tooth positions. The occlusal plane shows up to two tooth crown sufaces arranged labiolingually at mid-length of the dental battery, although one tooth is present throughout most of the length of the dental battery. There is a prominent single median ridge on the labial side of maxillary teeth (Fig. 5D). As preserved, the margins of the exposed crowns do not allow observing marginal papillae. The more complete and exposed maxillary teeth have a height/width ratio of 1.5; however, this value is probably higher given that part of those crowns are concealed by the ventral margin of the lateral surface of the maxilla.

### Postcranial elements

**Figure 6:**
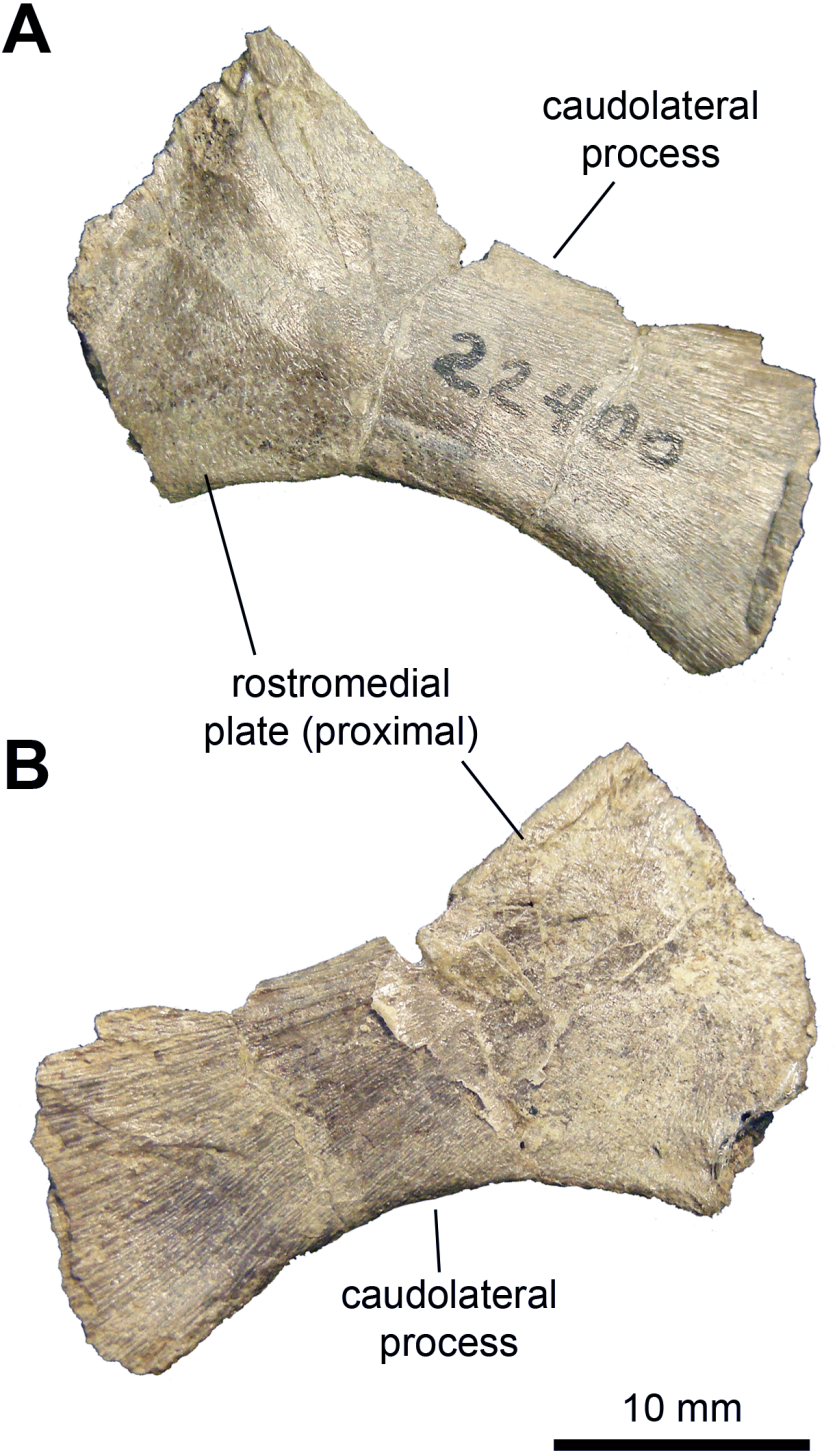
Axial element of *Maiasaura peeblesorum* perinates (YPM-PU 22400). (A & B) Partial left sternal plate in ventrolateral and dorsomedial views, respectively. Photographs by Albert Prieto-Marquez.

*Sternal plate:* This bone is only known from a single specimen preserving most of the caudolateral process and the base of the craniomedial plate (Fig. 6 and Table 2). The preserved craniomedial plate shows a slightly convex lateral surface and a flat medial face. The caudolateral process is missing its distal end. It is mediolaterally compressed and its medial surface displays a series of fine striations. The ventral margin of the sternal plate exhibits a more convex profile than that of the dorsal border.

**Table 2:** Selected measurements (in mm) of the axial, pectoral girdle and forelimb of the YPM-PU 22400 perinatal specimens of *Maiasaura peeblesorum*. Scapular and humeral measurements are taken from the most complete scapula (Fig. 7C) and humerus (Figs. 8C and 8D), respectively. Forerarm and manual measurements are from the articulated forearm and hand shown in Figs. 9E and 9F. All other pectoral and forelimb elements are of similar size to those measured below.

**Element**	**Measurement**
Sternal plate, length of preserved fragment	30
Sternal plate, minimum width of caudolateral process	10
Coracoid, length from the cranial end of the scapular facet to apex of ventral process (parallel to lateral margin of glenoid facet)	20.7
Coraoid, width from lateral margin of biceps tubercle to lateral margin of glenoid facet	15.1
Coracoid, length of glenoid facet	8.5
Coracoid, length of scapular facet	8.2
Scapula, length (parallel to dorsal margin) from cranial end of pseudoacromion process to incomplete distal end of scapular blade	66.5
Scapula, minimum width of proximal constriction	13.2
Scapula, maximum width of incomplete distal end of scapular blade	18.4
Humerus, length from dorsal margin of articular head to radial condyle	76.3
Humerus, length of deltopectoral crest	31.2
Humerus, maximum width from caudal margin to lateral margin of deltopectoral crest	15.4
Humerus, minimum diameter of shaft	8.7
Humerus, width of proximal margin	15.8
Humerus, width across distal condyles	16.6
Ulna, length	75.2
Ulna, mimimum diameter of shaft	5.6
Ulna, maximum depth of proximal end	11.3
Radius, length	71.6
Radius, mimimum diameter of shaft	4.5
Metacarpal III, length	35.5
Metacarpal IV, length	34.7
Metacarpal V, length	11.3
Phalanx III-1, length	10.2
Phalanx IV-1, length	10.4
Phalanx V-1, length	7.9

**Figure 7:**
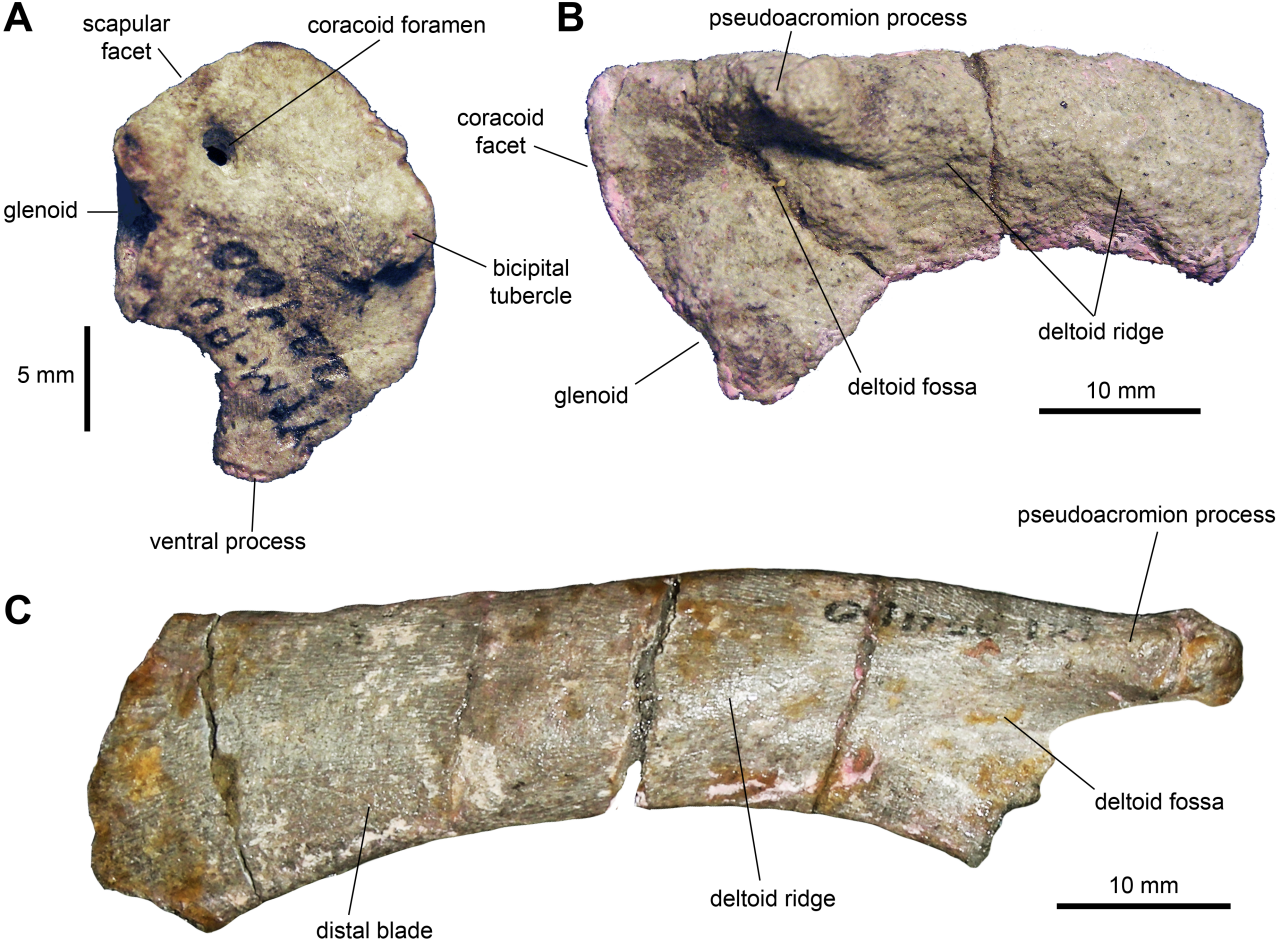
Pectoral girdle of *Maiasaura peeblesorum* perinates (YPM-PU 22400). (A) Right coracoid in lateral view. (B) Partial left scapula in lateral view. (C) Partial right scapula in lateral view. Photographs by Albert Prieto-Marquez.

*Coracoid:* The single known coracoid is mediolaterally compressed but complete (Fig. 7A and Table 2). It has a short, ventral process that is ventrally directed and comprises approximately one third of the length of the entire element. The scapular and glenoid facets are approximately 120 degrees from each other. The humeral facet is better developed than the scapular facet (which may be due to preservation), though both are approximately equal in length. The humeral facet is oval in articular view, deeply concave, and wider than the scapular facet. The latter is mediolaterally thin and flat. The coracoid foramen is relatively narrow, accounting for only one tenth of the length of the coracoid. This foramen perforates the dorsal region of the coracoid near the scapular facet. The craniolateral surface of the coracoid is convex and displays a broad biceps tubercle that does not project far beyond the craniodorsal margin.

*Scapula:* The available scapulae preserve most of the distal blade, deltoid ridge, glenoid and coracoid facet (Figs. 7B and 7C; Table 2). Near the dorsal margin of the proximal region, the pseudoacromion process projects laterally and displays a mostly horizontal long axis, except rostrally where it is slightly curved dorsally along with the dorsal margin of the scapula. The deltoid ridge is moderately developed and originates from the caudal extent of the pseudoacromion process. There is a deep deltoid fossa ventral to the pseudoacromion process. The coracoid facet comprises up two-thirds of the proximal margin. This facet is crescent-shaped in articular view and is approximately three times the length of the glenoid. The glenoid forms one-third of the proximal margin of the scapula and is oval in articular view. Both the coracoid and glenoid facets are slightly concave. The proximal constriction of the scapula is tear shaped in cross section, wider on the dorsal side. From this constriction, the scapular blade thins mediolaterally. The distal end of the scapular blade is incompletely preserved. Distally, however, the blade does not expand substantially relative to the proximal constriction (Fig. 7C). Specifically, at its distal-most extent the blade is only 1.35 times deeper than the proximal constriction. The scapular blade is medially curved towards the distal tip. The medial surface of the scapula is smooth and flat.

**Figure 8:**
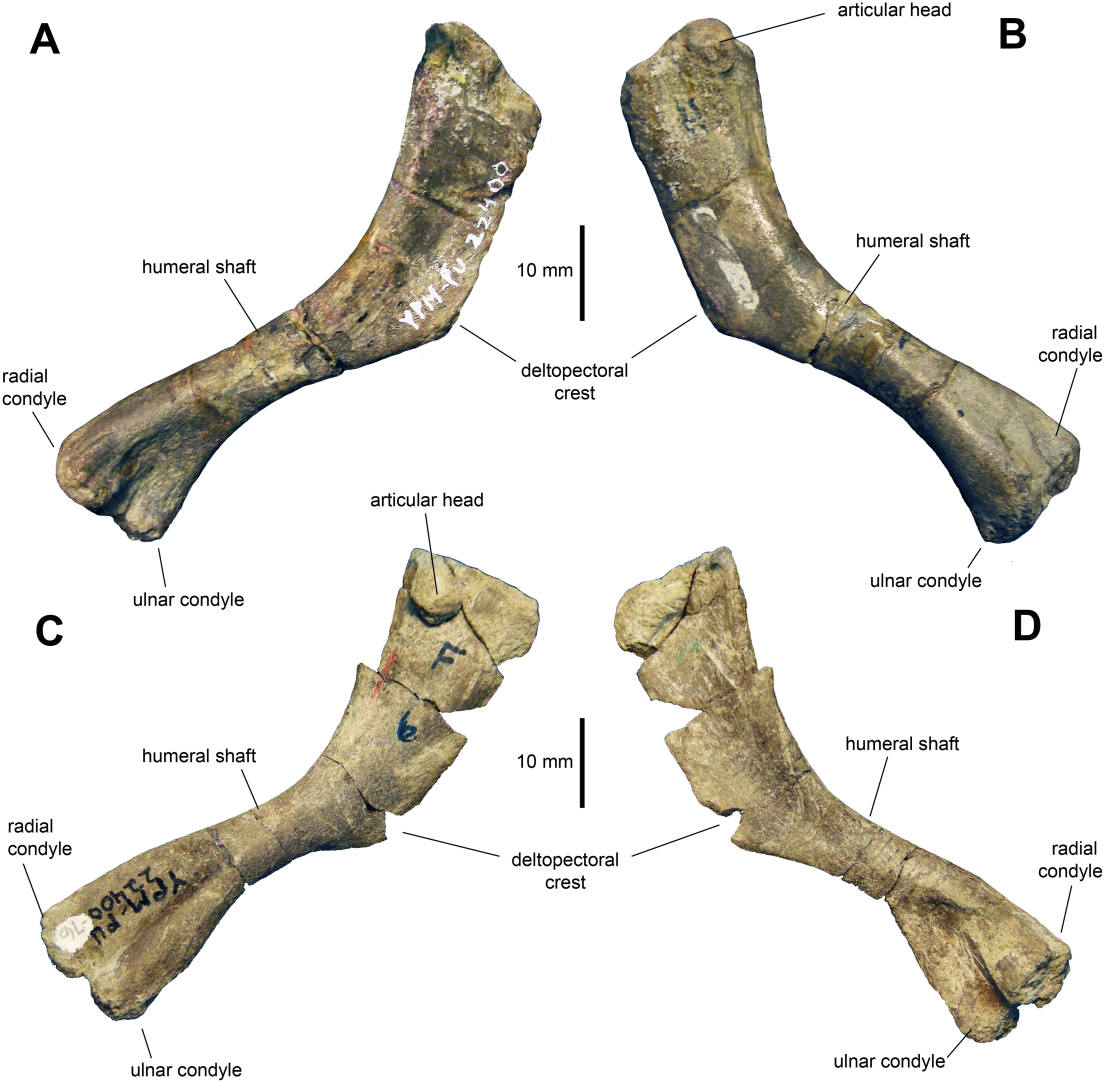
Humeri of *Maiasaura peeblesorum* perinates (YPM-PU 22400). (A) and (B) Left humerus in craniomedial and caudolateral views, respectively. (C) and (D) Right humerus in caudolateral and craniomedial views, respectively. Photographs by Albert Prieto-Marquez.

*Humerus:* The humerus (Figs. 8A–8D and Table 2) is a gracile bone, with expanded proximal and distal ends. The central shaft is 45 percent of the width of the proximal margin of the humerus. The deltopectoral crest is slightly less than half of the length of the humerus. The humerus reaches a maximum breadth of 1.65 times the minimum width of the humeral shaft at three quarters of the length of the deltopectoral crest. The proximal end of the humerus flares moderately. The articular head is prominent and forms one third of the width of the proximal margin. There is a low ridge that extends from the humeral head distally along the caudolateral margin of the element. The shaft is oval in cross section at mid-length. The two distal condyles are approximately equal in length and width.

**Figure 9:**
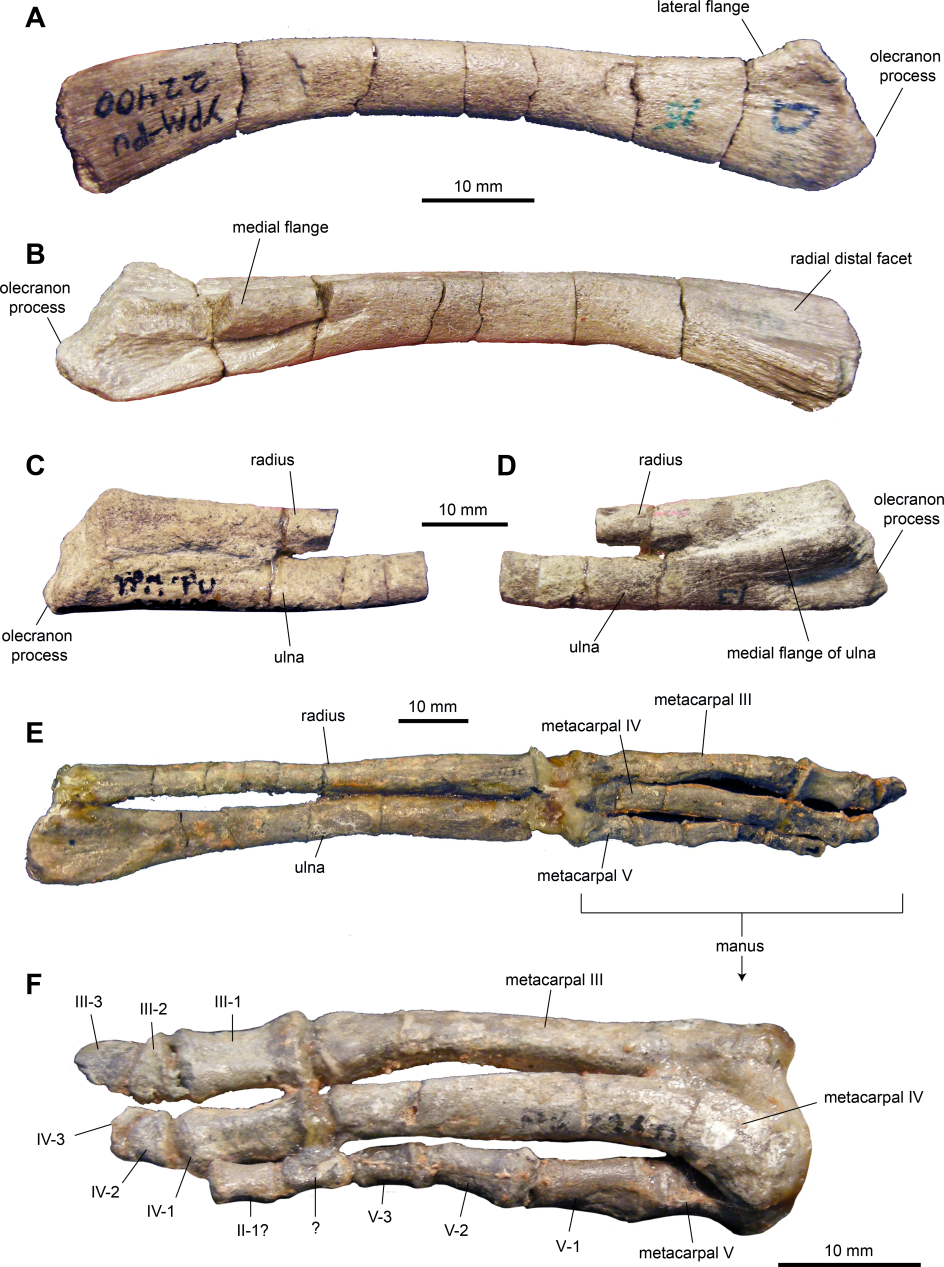
Forelimb elements of *Maiasaura peeblesorum* perinates (YPM-PU 22400). (A) and (B) Left ulna in lateral and medial views, respectively. (C) and (D) Proximal regions of articulated right ulna and radius in lateral and medial views, respectively. (E) Articulated left ulna, radius, and manus in ventral view. (F) Detail of the manus shown in (E), in dorsal view. Photographs by Albert Prieto-Marquez.

*Ulna*: The ulna (Figs. 9A–9D and Table 2) has a shaft that is ten times longer than it is deep at mid-length. The shaft is curved cranioventrally, particularly along its ventral margin, and becomes deeper both proximally and distally. Proximally, the element is triangular in cross section with a broad and prominent olecranon process. The lateral and distal flanges are moderately developed. Distally the ulna becomes gradually more oval in cross section.

*Radius:* The radius (Figs. 9C–9E and Table 2) is rod-like and slender, approximately 16 times longer than it is deep at mid-length. The bone is slightly expanded proximally and distally. At mid-length, the shaft is oval in cross section. The proximal end is subtriangular in proximal view. The distal third of the radius is slightly compressed dorsoventrally.

*Metacarpals:* No metacarpal II was identified in the sample of specimens. Metacarpals III and IV are elongate and slender bones (Figs. 9E and 9F; Table 2). Metacarpal III is slightly longer than metacarpal IV, being approximately nine times longer than it is wide at mid-length. Metacarpal III shows a flat dorsal surface and medial and lateral surfaces that converge medioventrally to produce a triangular cross section along the mid segment of the element. The bone is slightly expanded at its proximal and distal ends. The proximal end shows a subtriangular profile, whereas the distal end is trapezoidal with a wider dorsal end.

Metacarpal IV is slightly thicker than metatarsal III and eight times longer than it is wide at mid-length. It is characterized by a proximal end that is mediolaterally expanded, dorsoventrally compressed, and laterally deflected. Distally, metacarpal IV becomes mediolaterally compressed.

Metacarpal V is subconical in shape and approximately 30 percent of the length of metacarpals III and IV. The distal portion is twisted dorsolaterally relative to the proximal region. The dorsal surface is proximodistally concave and mediolaterally convex proximally and distally.

*Manual phalanges*: Proximal phalanges III-1 and IV-1 are subrectangular elements that are dorsoventrally compressed and slightly constricted mediolaterally at mid-length (Figs. 9E and 9F; Table 2). A phalanx II-1 is possibly present in the sample and displays a blocky subrectangular morphology, gently constricted mediolaterally along its middle section. This element is substantially smaller than phalanges III-1 and IV-1. Subsequent more distal phalanges III-2 and IV-2 are proximodistally compressed and wedge-shaped. Unguals III-3 and IV-3 are dorsoventrally compressed and D-shaped elements. On digit V, phalanges are subconical in overall morphology and, distally, they become progressively shorter and thinner.

**Figure 10:**
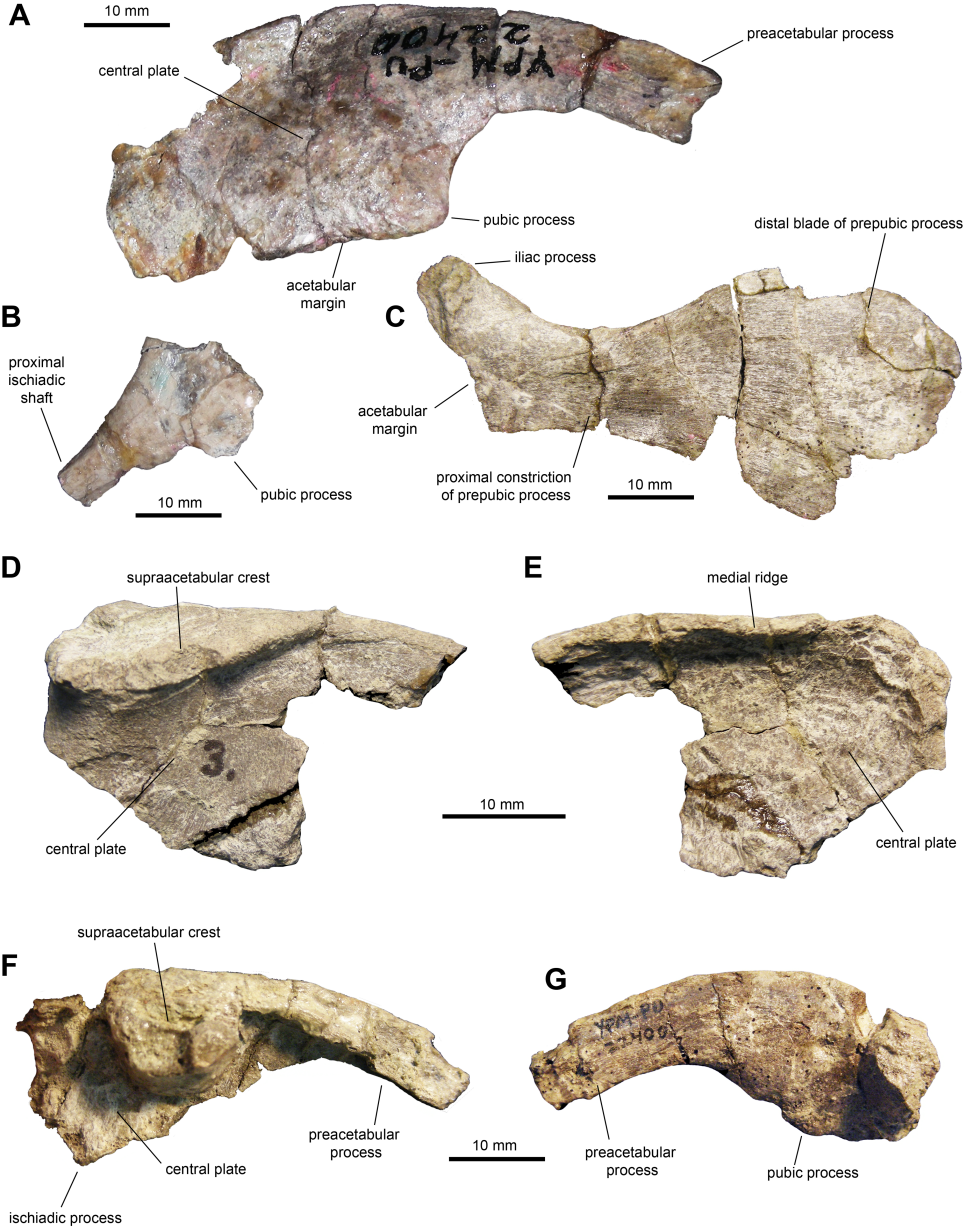
Pelvic elements of *Maiasaura peeblesorum* perinates (YPM-PU 22400). (A) Right ilium in lateral view. (B) Partial proximal region of right ischium in lateral view. (C) Right pubis in lateral view. (D & E) Partial central plate of right ilium in lateral and medial views, respectively. (F & G) Partial central plate and preacetabular process of right ilium in lateral and medial views, respectively. Photographs by Albert Prieto-Marquez.

*Ilium*: This pelvic bone is known from a few partial specimens (Figs. 10A, 10D–10G; Table 3). The preacetabular process is strongly compressed mediolaterally and projects cranioventrally forming a 150-degree angle with the dorsal margin of the supraacetabular crest. The medial surface of the central plate is flat and its dorsal margin forms a strong medial ridge for reception of the transverse process of the sacral vertebrae. The supraacetabular crest shows an asymmetrical, caudally skewed U-shaped lateral profile. This structure is cranioventrally extensive, ending cranially next to the proximal region of the preacetabular process. The ventral margin of the supraacetabular crest extends to about one third of the depth of the central iliac plate. The ischiadic and pubic processes, along with the acetabular margin that they enclose, are incompletely preserved. The postacetabular process is missing in all the available ilia.

**Table 3:** Selected measurements (in mm) of pelvic and hindlimb elements of the YPM-PU 22400 perinatal specimens of *Maiasaura peeblesorum*. Measures of the ilium, fibula and pedes are taken from the specimens shown in Figs. 10A, 11E–11H, and 12F–12G, respectively.

**Element**	**Measurement**
Ilium, length from incomplete tip of preacetabular process to caudal end of iliac central plate	60.1
Ilium, depth from dorsal-most apex of central plate to pubic process	22.6
Pubis, length from distal margin of distal blade to acetabular margin	57.4
Pubis, minimum width of proximal constriction	12.2
Pubis, maximum depth of distal blade (estimated)	30
Femur, mean length of 24 femora	138
Tibia, mean length of 16 tibiae	124
Fibula, length	109
Astragalus, mediolateral width	23
Calcaneum, craniocaudal width	12.9
Metatarsal II, length	42.1
Metatarsal III, length	51.7
Metatarsal IV, length	41.5
Phalanx II-1, length	11.5
Phalanx III-1, length	20.3
Phalanx IV-1, length	11.1

*Ischium*: All that is preserved from this element are a few fragmentary proximal regions with partial iliac or pubic processes (Fig. 1B), along with fragments of shafts. The iliac process is subrectangular in lateral and medial views, mediolaterally compressed and slightly expanded distally. The iliac process is 2.65 times longer than it is craniocaudally wide at mid-length. The distal facet displays an oval outline. The pubic process is mediolaterally compressed and incompletely preserved.

*Pubis*: The pubis is known from a single element missing the postpubic process and dorsal and ventral portions of the cranial region of the distal blade of the prepubic process (Fig. 10C and Table 3). At the proximal end of the pubis, the iliac process extends caudodorsally to contribute dorsally to the acetabular margin, forming a 140-degree angle with the longitudinal axis of the prepubic process. This subtetrahedral process is slightly compressed mediolaterally (which could be a preservational artifact) and is missing its ventral extent. The maximum ventral concavity of the prepubic process is symmetrically located below the maximum dorsal concavity. The prepubic process has a relatively deep and paddle-shaped distal blade. At its deeper point, the blade is 2.5 times wider than the proximal constriction. Taking into account the missing portions of the cranial region of the distal blade, it appears that the cranioventral region was more prominent than the craniodorsal one; thus, the blade was likely deflected cranioventrally.

**Figure 11:**
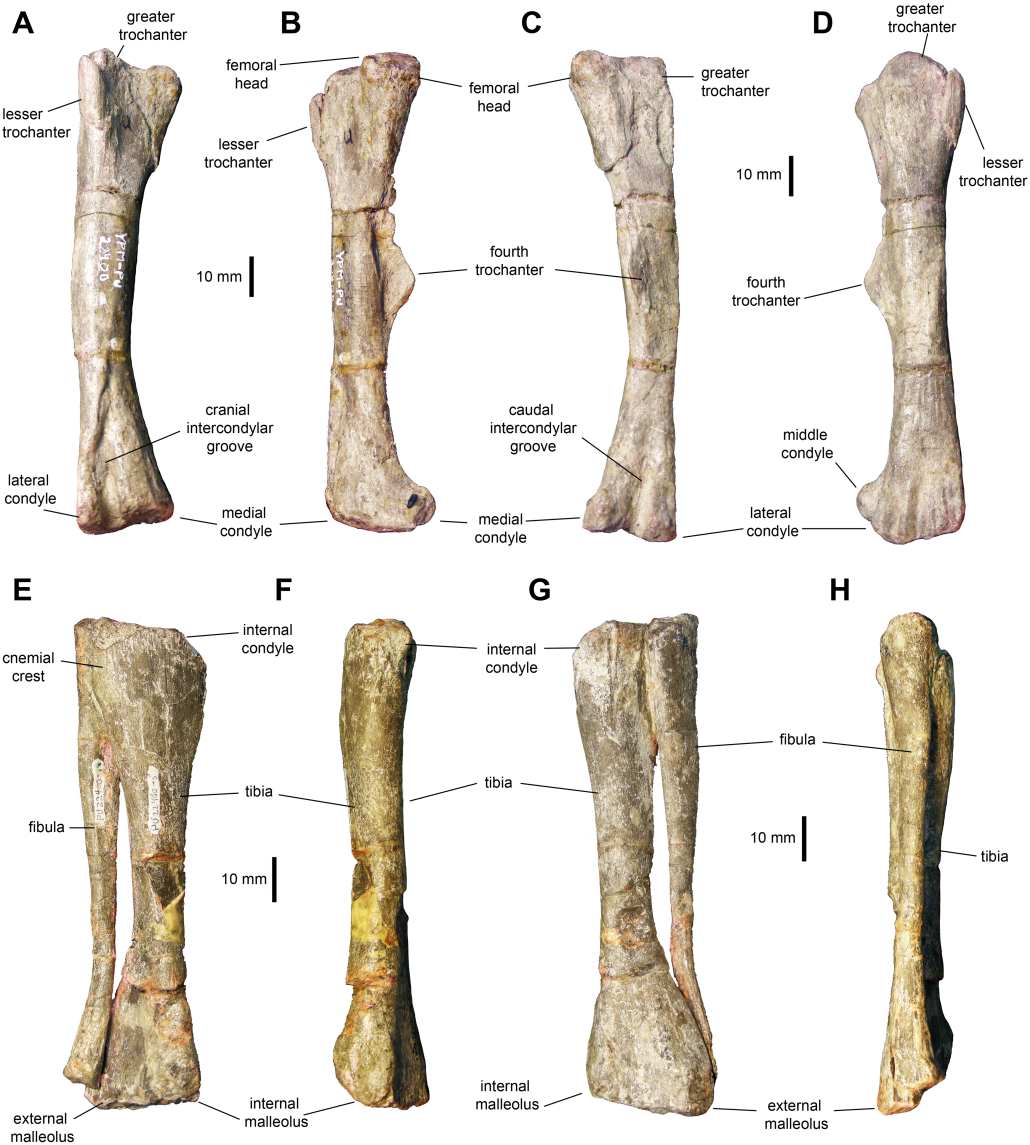
Hindlimb elements of *Maiasaurapeeblesorum* perinates (YPM-PU 22400). (A–D) Right femur in cranial, medial, caudal, and lateral views, respectively. (D–G) Articulated right tibia and fibula in cranial, medial, caudal, and lateral views, respectively. Photographs by Albert Prieto-Marquez.

*Femur*: The femur (Figs. 11A–11D and Table 3) displays a well-developed greater trochanter that is, however, narrower and more oval than the femoral head. The femoral shaft appears straight in lateral and medial views, but it shows a slight curvature in cranial and caudal views (Figs. 10A and 10C). The shaft is oval in cross section with the long axis oriented mediolaterally. The fourth trochanter forms an obtuse triangle projecting from the medial surface of the shaft. This process is mediolaterally thin and accounts for one fourth of the length of the femur. Distally, the medial and lateral condyles are slightly disproportionate, with the medial condyle reaching a greater width cranially and the lateral condyle being broader caudally.

**Figure 12:**
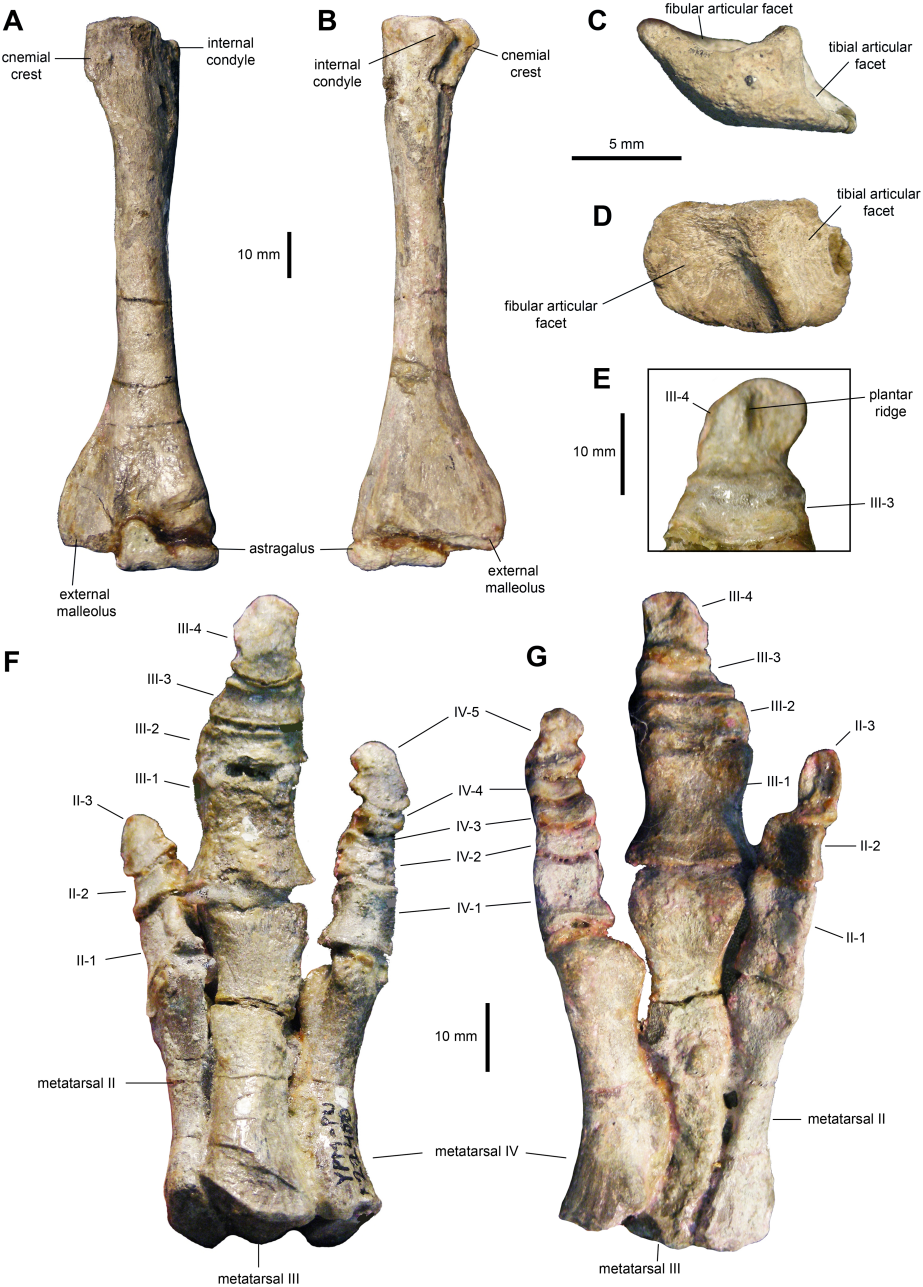
Hindlimb elements of *Maiasaura peeblesorum* perinates (YPM-PU 22400). (A & B) Left tibia and astragalus in cranial and caudal views, respectively. (C & D) Left calcaneum in lateral and dorsal views, respectively. (E) Distal pedal digit III of the left pes, showing a ridge on the plantar surface of the ungual. (F & G) Right pes in dorsal and plantar views, respectively. Photographs by Albert Prieto-Marquez.

*Tibia*: The tibia (Figs. 11E–11H, 12A and 12B; Table 3) features a straight shaft that narrows at the distal three fifths of its length, showing an oval cross section. The bone is greatly expanded craniocaudally at its proximal end and mediolaterally at its distal end. At the proximal region, the tibia has a prominent internal condyle. The cnemial crest is well-pronounced and extends along the cranial surface of the diaphysis to wrap around the proximal end of the fibula. The proximal articular surface is crescent-shaped. At the distal end, the external malleolus projects further distally than the internal malleolus.

*Fibula:* The fibula shows a relatively slender shaft that is craniocaudally expanded proximally and distally (Figs. 11E–11H; Table 3). The proximal margin of the fibula is gently crescentic. Distally, the shaft gradually becomes thinner, reaching the narrowest point near three quarters of its length, before widening again towards its contact with the calcaneum.

*Proximal tarsals*: The astragalus is a saddle-shaped element that receives slightly more than the medial half of the ventral surface of the tibia (Figs. 12A and 12B). Its cranial surface is triangular, with a laterally skewed outline. The ascending process comprises one third of the width of the astragalus. The process is twice as high as the main body of the astragalus. The caudal surface of the atragalus is substantially shallower and displays the outline of an obtuse triangle.

The calcaneum is a crescent-shaped and relatively small element in the proximal tarsus (Figs. 12C and 12D). Its ventral surface is craniocaudally convex, whereas its dorsocaudal side is strongly concave. The latter contains a tall, thick and oblique ridge that separates the D-shaped articular facets for the fibula and the tibia. The medial surface of the calcaneum is substantially shallower than the lateral side.

*Metatarsals*: These are robust and tightly appressed elements in the pes (Figs. 12F and 12G; Table 3). Metatarsal II is 75 percent and nearly 90 percent of the length of metatarsals III and IV, respectively. Metatarsal II is mediolaterally compressed and shows dorsoventrally expanded proximal and distal ends. The proximal end is 40 percent deeper than the distal end, and displays a lenticular proximal surface. The distal articular surface shows an asymmetrical, medially skewed subtrapezoidal outline with a deeply concave ventral margin.

Metatarsal III is slightly compressed dorsoventrally, reaching the most constricted point near its distal third. The proximal and distal ends are moderately expanded, particularly the former. The ventral surface of metatarsal III displays a shallow and long convexity from its mid-length to its proximoventral margin. The proximal facet shows a crescentic profile, caused by a deeply excavated medial surface of the proximal third of the metatarsal. The distal surface is subtrapezoidal, with a dorsal margin that is wider than the ventral border.

Metatarsal IV is also dorsoventrally compressed; however, in this element the point of maximum constriction occurs at mid-length. Both the proximal and distal ends are nearly equally deep. A thick flange occurs on the medial side near the proximal end of the metatarsal. Adjacent and proximal to this flange, the bone surface shows fine longitudinal striations. This medial surface is strongly concave perpendicularly to the striantions. The proximal surface is D-shaped, whereas the distal facet displayes a subtrapezoidal outline that is strongly skewed medially.

*Pedal phalanges*: The most proximal phalanges II-1, III-1 and IV-1 are blocky elements that are slightly longer than wide, gently constricted mediolaterally at mid-length (Figs. 12F and 12G; Table 3). Phalanx III-1 differes from II-1 and IV-1 in showing a substantially more mediolaterally expanded proximal margin and being approximately twice the size of the other two phalanges. More distal phalanges III-2 and III-3 are strongly compressed proximodistally, so that they are over three and two times wider than long, respectively. Phalanges distal to II-1 and IV-1 are also blocky, but relatively less abbreviated proximodistally than III-2 and III-3. Finally, pedal unguals are arrow-shaped elements that wedge distally in thickness. A median ridge is present on the plantar surface of the unguals (Figs. 12E and 12G), as it occurs in *Brachylophosaurus canadensis* and adult *Maiasaura peeblesorum* (Prieto-Márquez, 2005).

## Discussion

### Skeletal growth changes in *Maiasaura peeblesorum*

*Skull and mandible*: The available YPM-PU 22440 cranial elements provided an approximation of the proportions of the perinate skull and mandible. The perinate skull is characterized by a shorter pre-orbital region than in adults. The distance from the caudal margin of the rostral process of the jugal to an estimated position of where the oral margin of the premaxilla or predentary would be located (approximated by the position of the rostral end of the dentary), is about 40 percent of the basal skull length (from the tip of the dentaty to the caudal margin of the quadrate). In adults like TCMI 2001.89.2, this pre-orbital region accounts for 63 percent of the basal skull length. The perinate skull is also deeper than that of adults. This is evidenced by the fact that in perinates the dentary is only 20 percent longer than the quadrate, whereas in adults like TCMI 2001.89.2 the dentary is 50 percent longer than the quadrate. Furthermore, based on the width of the orbital margin of the jugal, it is estimated that the rostrocaudal diameter of the orbit is 35 percent of the basal skull length. In contrast, adults like OTM F138 display an orbital rostrocaudal diameter of only 20 percent of the basal skull length. This decrease in the relative size of the orbit during the ontogeny of *Maiasaura peeblesorum* has also been documented in other saurolophine (Maryanska & Osmólska, 1981; Prieto-Márquez, 2005) and lambeosaurine (Horner & Currie, 1994; Evans, Forster & Reisz, 2005) hadrosaurids, as well as in crocodilians (Dodson, 1975) and other dinosaurs (Varricchio, 1997).

The adult maxilla differs from that of the perinates in possessing a relatively shorter ectopterygoid shelf and less steep premaxillary shelf. Both of these ontogenetic changes were also reported in *Edmontosaurus annectens* (Prieto-Márquez, 2014). In adult *Maiasaura peeblesorum* (e.g., OTM F138) the ectopterygoid shelf accounts for one third of the length of the maxilla (excluding the rostromedial process) and the premaxillary shelf and rostromedial process form a 25 to 30-degree angle with the tooth row. A decrease of the steepness of the premaxillary shelf of the maxilla during ontogeny has also been reported the lambeosaurine *Hypacrosaurus stebingeri* (Horner & Currie, 1994) and it is probably related to the shorter rostrum in early ontogeny. The mid-point of the base of the dorsal process is slightly more rostrally positioned in perinates (Fig. 2A) in relation to adults (e.g., OTM F138). The ectopyerygoid shelf is remarkably inclined in adult *M. peeblesorum* (e.g., OTM F138), a condition shared with the other brachylophosaurins, such as *Brachylophosaurus canadensis* (Prieto-Márquez, 2005), *Acristavus gagslarsoni* (Gates et al., 2011: fig. 3B) and *Probrachylophosaurus bergei* (Freedman Fowler & Horner 2015: fig. 5C).

Adult jugals have slightly less disparity between the thickness of the caudal and rostral constrictions. Thus, in adults like OTM F138 and YPM-PU 22405 the caudal constriction is about 1.25 times thicker, whereas in perinates it is 1.3 times thicker. It is worth noting that there is substantial variation among adults in the degree of caudal inclination of the postorbital process of the jugal. In some exemplars like TCMI 2001.89.2, the postorbital process is more inclined (170 degrees with the long axis of the rostral process) than in the perinates (140 degrees, as indicated above), whereas in other specimens like OTM F138 the process is less tilted (120 degrees). This variation among adults is translated to the disparity in width between the orbital and infratemporal margins of the jugal: in those adult specimens where the postorbital process is more inclined caudally, the orbital margin becomes substantially wider than the infratemporal margin. The caudoventral flange is slightly less prominent in perinates, where it reaches a depth 1.57 times that of the caudal constriction. In the most complete adult jugal observed (TCMI 2001.89.2), this flange projects caudoventrally to reach a depth that is over 1.65 times the depth of the caudal constriction. The rostral process of the adult jugal exhibits a triangular caudoventral margin, whereas this projection is absent in the shallow caudoventral margin of the rostral process of the perinate.

The quadrate of the perinate has a distal lateral condyle that is relatively less expanded craniocaudally than that of the adult (e.g., OTM F138). In adults, there is a squamosal buttress on the caudal margin near the articular head (e.g., YPM-PU 22405). This buttress is not observed in the perinate. No ontogenetic changes were identified in the quadratojugal notch: in both perinates and subadults the notch occuppies 45 percent of the length of the quadrate; the mid-length of the notch is located ventral to the mid-length of the quadrate, specifically at a point found at nearly 60 percent of quadrate length.

As it occurs in other hadrosaurids (Campione & Evans, 2011; Bell, 2011; Prieto-Márquez, 2014), as well as in hadrosauroid outgroups (Prieto-Márquez, 2011), the dentary experienced an elongation of the mandibular ramus during growth. This is evidenced by the shallower and longer rami of juveniles like MOR 547 and adults such as YPM-PU 22405 and OTM F138. Likewise, the symphyseal process becomes longer; the proximal horizontal portion of the edentulous margin that precedes the sloping dorsal edge of the symphyseal process, equals to approximately one fifth of the length of the dental battery in adults. In the perinate, this margin is nearly absent (Fig. 4E) and the adjacent sloping border is much steeper, approaching a subvertical orientation in medial view, compared to adults. However, the ventral margin of the symphyseal process is similarly deflected in both adults (25-degree angle with the tooth row in YPM-PU 22405 and ROM F138) and perinates. The coronoid process is vertically oriented in those perinatal dentaries where this feature is preserved, whereas in adults it is tilted rostrally as it commonly occurs in hadrosaurids (Prieto-Márquez, 2010). This change in orientation of the coronoid process has also been reported in the saurolophine hadrosaurid *Saurolophus angustirostris* (Maryanska & Osmólska, 1981).

The perinatal dentition shows shorter tooth crowns in both the dentary and maxillary dental batteries. Specifically, in adult dentaries the height/width ratio of tooth crowns is 3.2–3.4 (Prieto-Marquez, 2008; e.g., OTM F138), whereas perinate crowns are nearly twice as tall as they are wide. This variation is associated with the increase in alveolar positions (and number of teeth in all the dimensions of the dental battery) that takes place during ontogeny in *Maiasaura peeblerosum*, and in hadrosaurids in general (Weishampel, Norman & Grigorescu, 1993). In the case of *M. peeblesorum*, adult dentaries show a tooth count of up to 38 positions (Prieto-Marquez, 2008). Adult *M. peeblesorum* teeth lack the fine accessory ridges oberved in the dentary teeth of the perinates. In the occlusal plane, three and two teeth arranged labiolingually become prevalent along much of the adult dental batteries of the denatry and maxilla, respectively, as in all other hadrosaurids (Horner, Weishampel & Forster, 2004).

There is threefold increase in the number of maxillary tooth positions from perinates (maximum of 15) to adults (e.g., 45 in OTM F138). As the number of alveoli increases during development, tooth crowns become mesiodistally narrower. However, as in perinates, adult maxillary tooth crowns show a prominent single median ridge with minute marginal papillae (e.g., ROM 44770).

*Postcranium*: The sternal plate displays a dramatic increase in the length of the caudolateral process in adults. Specifically, in perinates this process is about 2.5 times longer than wide at its minimum width (Fig. 6), whereas in adults the process is over fives times longer than wide at mid-length (e.g., TCMI 2001.89.2).

In the pectoral girdle, the minimum width of the proximal constriction of the scapula is 53 percent of the maximum depth of the proximal articular region (measured from the ventral apex of the glenoid perpendicular to the dorsal margin of the psudoacromion process). In adults, the minimum breadth of the proximal constriction is greater, reaching over 60 percent of the depth of the proximal region (e.g., TCMI 2001.89.2). A relatively thicker proximal constriction in adults was also reported in the lambeosaurine *Hypacrosaurus stebingeri* (Horner & Currie, 1994). The deltoid ridge becomes more prominent and demarcated in adults (e.g., ROM 44770). In the perinate, the dorsal and ventral margins of the scapular blade remain nearly parallel for a substantial part of its length, until reaching the distal extent of the blade where it shows a noticeable expansion (1.35 times the depth of the proximal constriction as preserved in YPM-PU 22400). In contrast, in adults like ROM 44770, the scapular blade expands continuously throughout its length until reaching a distal depth that is nearly 1.9 times the depth of the proximal constriction, similar to what was observed by Guenther (2014).

The deltopectoral crest of the adult humerus in *Maiasaura peeblesorum* accounts for half of the length of the humerus (e.g., TCMI 2001.89.2), in contrast to the slightly shorter perinate deltopectoral crest. The humeral shaft becomes more robust in adults, as noted by Dilkes (2001); for example, in TCMI 2001.89.2 the total humerus length/minimum shaft diameter ratio is 6.9, whereas in the perinates this ratio ranges from 8 to 9. However, this trend is not common to all hadrosaurids. For example, Horner & Currie (1994) reported a reduction in robutness during ontogeny in the lambeosaurine *Hypacrosaurus stebingeri*. During ontogeny there is an increase in the prominence and relative size of the humeral heads and tuberosities, such as that on the caudal surface of the humerus, as noted by Brett-Surman & Wagner (2007) and Guenther (2014). Additionally, as also pointed out by Brett-Surman & Wagner (2007), the difference in size between the larger ulnar and the radial condyles becomes greater in adults. For example, on average, in perinates the ulnar condyle is 17 percent wider craniocaudally than the radial condyle, whereas in adults like TCMI 2001.89.2 the ulnar condyle is 25 percent broader.

The ulna becomes proportionately more elongated during ontogeny, as it occurs in other taxa. Thus, in perinates, the ulna is on average 10.6 times longer than it is dorsoventrally thick at mid-length, whereas in adults like TCMI 2001.89.2 the ulna nearly 13 times longer than thick. The medial and lateral flanges of the proximal region of the ulna become more prominent in adults, a change previously reported also in *Edmontosaurus annectens* (Prieto-Márquez, 2014). As noted by Dilkes (2001), the olecranon process is less prominent in the perinates, a change that has also been reported in other hadrosaurids (Brett-Surman & Wagner, 2007; Prieto-Márquez, 2014).

In the manus, the perinatal metacarpals possess relatively less expanded distal and, particularly, proximal ends than those of adult specimens such as TCMI 2001.89.2. This variation has also been observed in *Edmontosaurus annectens* (Prieto-Márquez, 2014).

In the pelvic girdle, we observed a trend noted by Brett-Surman & Wagner (2007), who indicated that later in ontogeny the preacetabular process of the ilium becomes substantially thicker mediolaterally (e.g., ROM 44770). The pubis preserves the overall oval paddle-shape of the distal blade of the prepubic process, with a more prominent cranioventral margin that is possibly also present in perinates. However, while in perinates and juveniles like MOR 547 the maximum concave point of the dorsal margin of the proximal constriction of the prepubic process lie approximately above the ventral maximum concave point, in adults like ROM 44770 the concave ventral profile is cranially offset relative to the dorsal border.

In the hindlimb, the perinate femora display a slender shaft and less prominent distal condyles than adults like TCMI 2001.89.2. These ontogenetic changes have been previously identified among hadrosaurids (Grigorescu & Csiki, 2006; Guenther, 2014; Prieto-Márquez, 2014). The fourth trochanter also becomes more developed in adults, as previously noted by Dilkes (2001) and Guenther (2014), as it occurs in other hadrosaurids (Brett-Surman & Wagner, 2007). The cnemial crest of the tibia becomes more craniolaterally expanded in adults, as reported by Dilkes (1996). This variation was also observed in other hadrosaurids (Brett-Surman & Wagner, 2007). No substantial changes were evident in the pes, aside from an overall increase in robustness.

### Ontogenetic variation of phylogenetically informative characters in hadrosaurids

The following cranial characters, commonly used in inferring hadrosaurid evolutionary relationships (e.g., Horner, Weishampel & Forster, 2004; Gates et al., 2011; Freedman Fowler & Horner, 2015; Prieto-Márquez, Erickson & Ebersole, 2016; Prieto-Márquez & Gutarra, 2016), are not variable throughout the ontogeny of *Maiasaura peeblesorum*: presence of a rostromedial process in the maxilla; having a subtriangular joint surface for the jugal that is more laterally than dorsally-facing, with a lateroventrally-directed pointed corner that is located adjacent and slightly dorsal to the proximal end of the lateral ridge of the ectopterygoid shelf; six or less maxillary foramina reduced in number and forming either a row or cluster that is oriented rostrodorsally; large rostral maxillary foramen exposed laterally and opening near the dorsal margin of the premaxillary shelf; presence of maxilla-lacrimal contact; dorsoventrally thick continuous ectopterygoid ridge, becoming gradually thicker caudally; arcuate row of alveolar foramina above the mid-dorsoventral depth of the maxilla; rostral process of the jugal with caudoevntral apex located ventral to the caudal margin of the lacrimal process; degree of curvature of the caudal margin of the quadrate; position of the quadratojugal notch along the length of the quadrate (measured as the ratio between the distance from the mid-length of the notch to the qudadrate head and the dorsoventral length of the element); orientation of the dorsal margin of the quadratojugal notch of the quadrate (measured as the angle between this and the caudal margin of the element); morphology of the lateral profile of the quadratojugal notch of the quadrate; angle of deflection of the rostral ventral margin of the dentary; location of the origination of the ventral deflection of the dentary (measured as the ratio between the distance from the caudal margin of the coronoid process to the inflexion point of the ventral margin and the distance from the caudal margin the coronoid process to the rostralmost alveolus); having a coronoid process with well-developed expansion of both the caudal and, especially, the rostral margin; the presence of fine striations on the medial surface of the coronoid process, located near the caudal margin; well-developed expansion of the lateral side of the dentary ventral to the coronoid process, with an angle (between the lateral surface of the dentary and that of the region caudoventral to the coronoid process) up to 165 degrees; longitudinal axis of the dentary occlusal plane being parallel to the lateral side of the dentary; caudal end of the dental battery extending caudal to the coronoid process; the coronoid process laterally offset relative to the tooth row, with a concave platform separating the base of the process from the dental battery; position of the primary median ridge on the enameled lingual side of dentary and maxillary tooth crowns; size and morphology of marginal denticles in dentary and maxillary teeth; and concave occlusal surface of the dentary dental battery.

In the postcranium, characters in *Maiasaura peeblesorum* that can be regarded as invariable during ontogeny on the basis of the available data include: relative reduction in coracoid size relative to the size of the scapula; development and degree of curvature of the ventral process of the coracoid; length of the scapular blade relative to the length of the proximal region occupied by the deltoid fossa; subhorizontal orientation of the pseudoacromion process; lateroventral expansion of the deltopectoral crest of the humerus; angulation of the ventral margin of the deltopectoral crest; absence of manual digit I; elongation of the manus exemplified by elongation of metacarpals II through IV, measured as the ratio between the length of metacarpal III and its width of at mid-shaft; craniocaudal width, development and ventral projection of the supraacetabular crest of ilium; asymmetry of the lateral profile of the supraacetabular process; shape of the pubic process of the ilium; orientation of the dorsoventral expansion of the prepubic process of the pubis; depth and lateral profile of the distal blade of the prepubic process of the pubis; straight shaft of the femur; smooth and arcuate lateral profile of the fourth trochanter of the femur; tibia with cnemial crest further extended along the cranial surface of the proximal half of the diaphysis; absence of metatarsal I; length/width proportions of pedal phalanges; and presence of a plantar ridge on pedal unguals.

## Conclusions

The osteology of perinatal specimens of the saurolophine hadrosaurid *Maiasaura peeblesorum* is for the first time described in detail, offering insights into the morphology of the early ontogenetic stages in these animals. Most of the growth changes in cranial elements such as the maxilla and jugal are probably associated with the gradual elongation of the preorbital region of the skull during ontogeny. A similar trend occurs in the mandible, reflected in the longer dentaries of progressively larger specimens and the addition of teeth that show taller crowns. A number of phylogenetically informative characters of the maxilla, jugal, quadrate, dentary, teeth, as well as from the pectoral and pelvic girdles, and some limb bones, are invariable during ontogeny in *M. peeblesorum*. This indicates that specimens as immature as perinates may still provide useful (albeit limited) character data for phylogenetic inference in hadrosaurids.

## Institutional abbreviations

MOR: Museum of the Rockies, Bozeman, Montana, USA
OTM: Old Trail Museum, Choteau, Montana, USA
ROM: Royal Ontario Museum, Toronto, Ontario, Canada
TCMI: The Children’s Museum of Indianapolis, Indianapolis, Indiana, USA
YPM-PU: Yale Peabody Museum of Natural History, Princeton University collection, New Haven, Connecticut, USA

## Additional Information and Declarations

### Data Availability

The following information was supplied regarding data availability: The described perinatal material of *Maiasaura peeblesorum* is housed at the Yale Peabody Museum of Natural History, New Haven, Connecticut, USA.
